# Abatement of epileptic spike-wave discharges through single pulse stimulation

**DOI:** 10.1186/1471-2202-14-S1-P13

**Published:** 2013-07-08

**Authors:** Peter N Taylor, Yujiang Wang, Justin Dauwels, Gerold Baier

**Affiliations:** 1 School of Electrical & Electronic Engineering, Nanyang Technological University. Singapore; 2 Manchester Interdisciplinary Biocentre, University of Manchester, UK; 3 Centre for Organismal Studies, University of Heidelberg, Germany

## 

Spike-wave discharges (SWD) are a striking phenomena detectable on the electroencephalogram (EEG) of all patients during absence seizures. There is experimental and clinical evidence to suggest that seizures can be terminated early through the use of short auditory stimulation [1], however, stimulation protocols for seizure abatement are underdeveloped and their varied success is poorly understood.

**Figure 1:**
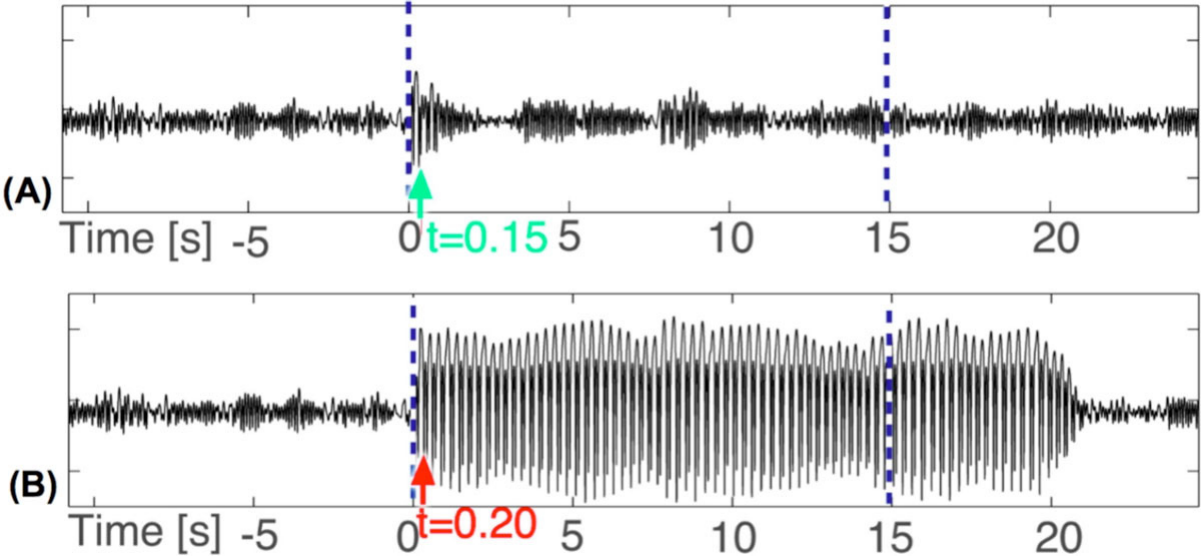
**(A) Successful single pulse stimulation applied at 0.15 seconds after seizure onset**. Blue dashed line indicates the SWD duration without a stimulus. (B) Unsuccessful single pulsestimulation applied 0.2 seconds after seizure onset.

In this work we extend the model of [2] to account for known thalamocortical connectivity which has previously been implicated in SWD [3]. This model is capable of producing transient spike-wave trains upon perturbation, for example, through the inclusion of noise. We show that a single pulse perturbation during a simulated seizure can, if applied with the correct timing and amplitude, successfully terminate the seizure early (Figure. 1a). Furthermore, if the same stimulus is applied incorrectly (e.g. at a different time) the seizure could be prolonged (Figure. 1b). The complex phase and amplitude dependency of successful stimulation can be explained in the model with its nontrivial phase space configuration. The complex and sensitive dependency could account for the variations in success of different clinical and experimental stimulation studies. Our modeling approach makes the prediction that these optimal stimuli can be predicted through the use of a learning algorithm included in a closed-loop stimulation device as suggested by [1]. Successful clinical implementation and application of such a learning algorithm could have dramatic impact on epileptic patients and offer a potential alternative to anti-epileptic drug based therapy. The combination of animal experiments on seizure control of SWD [4,5] and the current model predictions regarding strength and timing could lead to improved translation into the clinical setting.
